# Pseudomyxome péritonéal secondaire à une tumeur mucineuse de l’ovaire: à propos d’un cas

**DOI:** 10.11604/pamj.2019.33.283.17203

**Published:** 2019-08-02

**Authors:** Mohamed Said Belhamidi, Youssef Zorkani, Hicham Krimou, Abdessamad Kaoukabi, Mohamed Menfaa, Fouad Sakit, Karim Choho

**Affiliations:** 1 Service de Chirurgie Viscérale, Hôpital Militaire Moulay Ismail de Meknès, Meknès, Maroc; 2 Service de Psychiatrie, Hôpital Militaire Moulay Ismail de Meknès, Meknès, Maroc

**Keywords:** Pseudomyxome péritonéal, tumeur ovarienne mucineuse, cytoréduction, Pseudomyxoma peritonei, ovarian mucinous carcinoma, cytoreductive surgery

## Abstract

Le pseudomyxome péritonéal (PMP) ou l'ascite gélatineuse est une entité rare, décrite pour la première fois par R. Wyerth en 1884. Il correspond à une atteinte péritonéale diffuse, composée d'une ascite mucineuse et d'implants épithéliaux mucineux multifocaux. Cette maladie touche essentiellement les femmes. Son incidence est estimée à 2 cas par un million d'habitants. Le pseudomyxome péritonéal peut rester asymptomatique, découvert lors d'une laparotomie. Le symptôme le plus fréquent est représenté par une distension abdominale associée à des douleurs abdominales diffuses. Le scanner abdominal constitue le moyen diagnostique le plus spécifique. Il met en évidence des signes pathognomoniques de l'ascite gélatineuse. Les tumeurs mucineuses de l'appendice sont la cause la plus fréquente du pseudomyxome péritonéal avec 90% des cas. L'origine ovarienne en constitue une cause très rare. Il existe essentiellement deux types de traitement du PMP: premièrement le « debulking » chirurgical multiple et deuxièmement la chirurgie de cytoréduction avec chimiothérapie intra-péritonéale péri-opératoire consistant en une chimiothérapie hyperthermique intrapéritonéale avec ou sans chimiothérapie intrapéritonéale post opératoire immédiate. Nous rapportons un cas de pseudomyxome péritonéal secondaire à une tumeur mucineuse de l'ovaire gauche.

## Abstract (en)

Pseudomyxoma peritonei (PMP), also referred to as gelatinous ascites, is a rare disorder, described for the first time by R. Wyerth in 1884. It is characterized by diffuse peritoneal involvement, composed of mucinous ascites and multifocal mucinous epithelial implants. This disease mainly affects women. Its incidence is estimated at 2 cases per one million inhabitants. Pseudomyxoma peritonei can be asymptomatic, discovered during a laparotomy. The most common symptom is abdominal distension associated with diffuse abdominal pain. Abdominal CT scan is the most specific diagnostic tool. It shows pathognomonic signs of gelatinous ascites. Mucinous neoplasms of the appendix are the most frequent cause of pseudomyxoma peritonei accounting for 90% of cases. Pseudomyxoma peritonei of ovarian origin is very rare. There are essentially two types of treatment: multiple debulking surgery and cytoreductive surgery with perioperative intraperitoneal chemotherapy consisting of hyperthermic intraperitoneal chemotherapy with or without immediate postoperative intraperitoneal chemotherapy. We report a case of pseudomyxoma peritonei secondary to mucinous carcinoma of the left ovary.

## Introduction

Le pseudomyxome péritonéal ou maladie gélatineuse est une entité rare qui correspond à une atteinte péritonéale diffuse, composée d'une ascite mucineuse et d'implants épithéliaux mucineux multifocaux. Son incidence est de 2 cas par un million d'habitants. Le pseudomyxome péritonéal peut rester asymptomatique, découvert lors d'une laparotomie. Le scanner constitue le moyen diagnostique le plus spécifique. L'origine appendiculaire de l'ascite gélatineuse est la plus fréquente et représente 90% des causes. Les tumeurs mucineuses de l'ovaire en constituent une cause très rare. Nous rapportons un cas de pseudomyxome péritonéal secondaire à une tumeur mucineuse de l'ovaire gauche.

## Patient et observation

**Figure 1:**
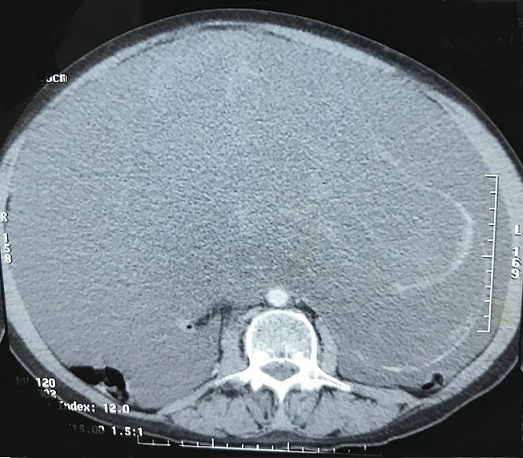
Coupe scannographique objectivant une ascite cloisonnée et hétérogène

**Figure 2:**
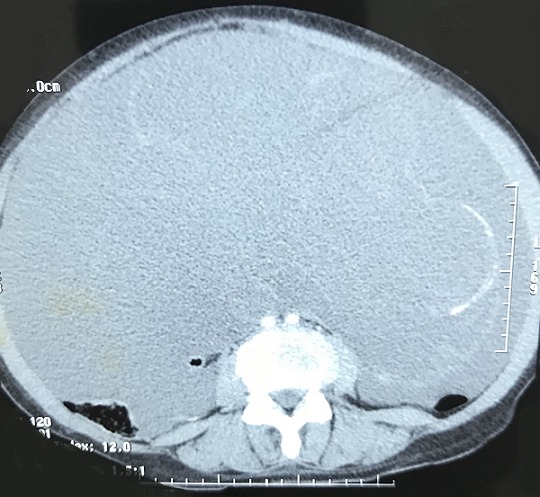
Tomodensitométrie abdominale montrant des calcifications sur les septas

**Figure 3:**
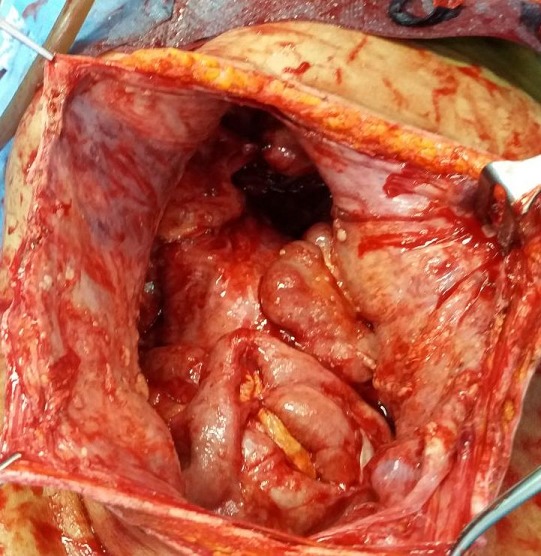
Laparotomie médiane: image d’implants mucineux péritonéaux

**Figure 4:**
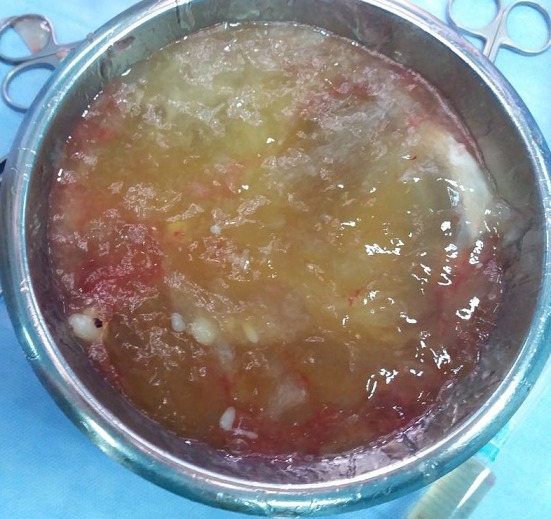
Liquide d’ascite gélatineuse évacuée

**Figure 5:**
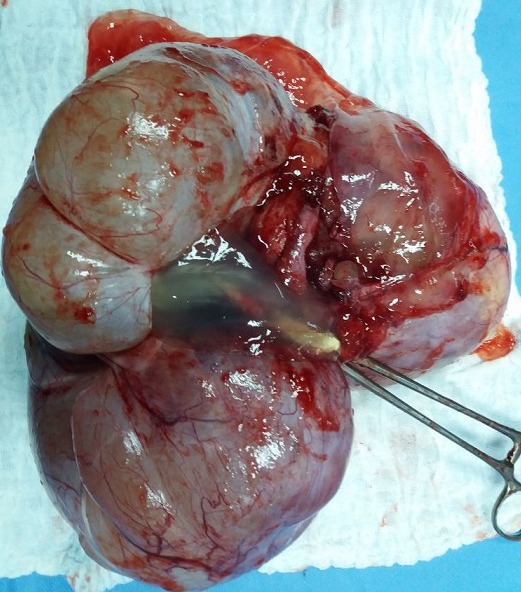
Pièce opératoire d’une annexectomie gauche, emportant la tumeur kystique de l’ovaire

Il s'agit d'une femme âgée de 51 ans, jamais opérée, connue hypertendue et équilibrée sous traitement médical. Le début de la symptomatologie remontait à 3 mois par l'installation des douleurs hypogastriques modérées, sans signes gynécologiques évidents, en particulier des métrorragies et des leucorrhées. La patiente ne présentait ni de troubles de transit intestinal ni de rectorragies. Par ailleurs, il n'y avait pas de signes généraux en faveur d'un syndrome infectieux. L'évolution clinique a été marquée par une distension abdominale avec altération de l'état général de la malade, ce qui a motivé son hospitalisation dans notre Service de Chirurgie Viscérale de l'Hôpital Militaire de Moulay Ismail de Meknès. L'examen physique général a mis en évidence des signes de déshydratation et de dénutrition, par ailleurs il n'avait pas de fièvre ni de pâleur cutanéomuqueuse. L'examen abdominal a objectivé une distension abdominale homogène et une matité diffuse de l'abdomen, sans circulation veineuse collatérale ni de masse palpable. Le reste de l'examen clinique est sans particularités. Le bilan biologique était normal en dehors d'une insuffisance rénale fonctionnelle: urée = 0,77g/l; créatinine sanguine = 10mg/l. Un cliché d'abdomen sans préparation a montré une grisaille abdominale diffuse, sans calcifications visibles. L'échographie abdominale a objectivé une ascite hétérogène de grande abondance avec des images hyperéchogènes sur la paroi abdominale. La tomodensitométrie (TDM) abdominale a mis en évidence une ascite de grande abondance, cloisonnée avec des calcifications sur les septa et une masse kystique hétérogène pelvienne de grande taille (15cm de grand axe) (Figure 1, Figure 2). Par ailleurs, pas d'autres lésions hépatiques, spléniques ou digestives n'ont été retrouvées. L'appendice n'était pas visible. Les marqueurs tumoraux, en l'occurrence l’antigène carcino-embryonnaire (ACE), l’antigène carbohydrate (CA) 19-9, étaient normaux. En raison d'un doute diagnostiqué, la décision d'une laparotomie exploratrice a été prise. L'exploration chirurgicale a mis en évidence une ascite gélatineuse de grande abondance et une grosse tumeur kystique au dépens de l'ovaire gauche avec des nodules blanchâtres disséminés sur le péritoine pariétal (Figure 3). Par ailleurs, il n'y avait pas de lésion appendiculaire, ni du tractus digestif ou autres lésions viscérales. On a évacué l'ascite (environ 13kg) (Figure 4) avec annexectomie gauche emportant la masse kystique (Figure 5). Une appendicectomie a été réalisée avec résection des implants péritonéaux de grande taille. Les suites opératoires étaient simples. L'étude anatomopathologique de la pièce opératoire était en faveur d'une tumeur mucineuse borderline de l'ovaire.

## Discussion

Pseudomyxome péritonéal ou maladie gélatineuse est une entité rare, décrite pour la première fois par R. Wyerth en 1884. Son incidence est estimée à 2 cas par un million d'habitants par an et 2 cas par 10000 laparotomies [1]. L'âge moyen de survenue est de 46 ans avec une atteinte préférentielle des femmes avec un sexe ratio d'un homme pour deux femmes. Notre cas était une femme âgée de 51 ans. Le pseudomyxome péritonéal correspond à une atteinte péritonéale diffuse, composée d'une ascite mucineuse et d'implants épithéliaux mucineux multifocaux, l'élément histologique fondamental est la présence de mucine extra-cellulaire dans la cavité péritonéale, pouvant être associée à des cellules épithéliales mucineuses, plus ou moins bien différenciées [2, 3].

Sur le plan clinique, la symptomatologie est non spécifique. Le pseudomyxome péritonéal (PMP) peut se révéler par des signes cliniques tardifs avec un état général souvent peu altéré. Ainsi le diagnostic est rarement fait avant la laparotomie [4]. Les signes révélateurs sont nombreux, dominés par l'augmentation progressive et isolée du volume abdominal et la douleur. Les autres signes fonctionnels sont principalement liés au retentissement de la maladie gélatineuse péritonéale sur le tractus digestif et/ou l'appareil urinaire [5]. Une étude de Esquivel et Sugarbaker portée sur 217 patients a retrouvé que les présentations cliniques ont été de 27% d'appendicite suspectée, 23% de distension abdominale progressive et 14% de hernie inguinale révélatrice. Parfois, le PMP est asymptomatique avec une découverte fortuite sur examen d'imagerie (échographie, TDM…) ou lors d'une laparotomie [6]. La chirurgie était longtemps considérée comme Gold standard du diagnostic selon Walensky *et al.* Notre patiente avait des douleurs abdominales atypiques avec une distension abdominale diffuse.

Sur le plan radiologique, le cliché de l'abdomen sans préparation (ASP) peut montrer une opacité prédominant d'un côté de l'abdomen qui est significative si elle est associée à des calcifications curvilignes épousant la périphérie de l'opacité [7]. Dans notre cas l'ASP n'était pas évocateur. En échographie comme en scanner, le diagnostic du pseudomyxome repose sur l'identification de trois lésions: l'ascite mucineuse et ses caractéristiques, les implants nodulaires péritonéaux s'ils sont visibles, et la tumeur primitive, qui n'est qu'exceptionnellement visualisée. L'ascite mucineuse du pseudomyxome est hétérogène et hypodense. Elle peut être cloisonnée et contenir de fines calcifications curvilignes. On peut retrouver un effet de compression extrinsèque sur le foie ou la rate « scalloping » et un refoulement des organes creux [8]. Chez notre patiente, la TDM a montré les mêmes signes radiologiques retrouvés dans la littérature, sans pouvoir localiser la tumeur primitive responsable de l'ascite. La TDM est particulièrement utile pour surveiller l'évolution de la maladie, et dépister une éventuelle récidive ou une complication (occlusion, abcès, compression urétérale avec dilatation de cavités excrétrices). Les marqueurs tumoraux demandés dans ce cadre sont le plus souvent l'ACE, le CA 19-9 et le CA125. Un taux sanguin d'ACE a été décrit dans la MGP associée aussi bien à une tumeur maligne qu'à une tumeur bénigne. Ces marqueurs, et surtout le CA 19-9, peuvent être utiles dans le suivi de la maladie et le diagnostic des récidives [9].

Un débat a longtemps subsisté sur l'origine du PMP: appendiculaire ou ovarienne. Une telle polémique existait car une atteinte simultanée de ces deux localisations était retrouvée chez la plupart des femmes. Grâce à l'immunohistochimie et au génie moléculaire, il est admis que l'origine est appendiculaire dans environ 90% des cas [10]. En 2002, Cornell *et al.* ont étudié l'expression du récepteur MCU 2 sur les cellules mucineuses péritonéale, leur positivité a confirmé que la tumeur primitive était une tumeur mucineuse appendiculaire. Selon Ronnett, Young et Prayson, les tumeurs mucineuses ovariennes de bas grade de malignité découvertes lors d'une MGP sont presque toujours des localisations secondaires d'une tumeur appendiculaire, surtout lorsqu'elles sont bilatérales (dans 32 à 80% des cas). Lorsqu'elles sont unilatérales, elles peuvent être des tumeurs primitives responsables du PMP et sont le plus souvent localisées à droite. Elles sont de grandes tailles et présentent souvent des implants ou des nodules à leur surface [11]. Notre patiente avait une grosse tumeur mucineuse de l'ovaire gauche sans atteinte appendiculaire. Ce cas est exceptionnellement retrouvé dans la littérature.

Il existe essentiellement deux types de traitement du PMP: le « debulking » chirurgical multiple et la chirurgie de cytoréduction avec chimiothérapie intra-péritonéale péri-opératoire: chimiothérapie hyperthermique intrapéritonéale avec ou sans chimiothérapie intrapéritonéale post opératoire immédiate [12]. Le but du « debulking » est d'enlever le maximum de gélatine et les formations tumorales. Les récidives symptomatiques se présentent sous forme d'occlusion intestinale, de douleurs abdominales ou de distensions abdominales, sont traitées par un nouveau « debulking » [13]. Chaque réintervention devient de plus en plus difficile.

La chirurgie de cytoréduction (CCR) consiste à reséquer toute formation tumorale visible en effectuant des gestes de péritonectomies. Une hémicolectomie droite et une hystérectomie avec salpingo-ovariectomie bilatérale chez la femme sont recommandées. Le but de la CCR est de reséquer toute tumeur visible en ne laissant que des nodules tumoraux de moins de 2,5mm de diamètre, et cela pour que la chimiothérapie intrapéritonéale périopératoire soit efficace [14]. Chez notre patiente, on a opté pour la technique de « debulking » avec une évacuation du maximum de gélatine sans chimiothérapie hyperthermique intrapéritonéale en raison de sa non disponibilité dans notre hôpital.

## Conclusion

Le pseudomyxome péritonéal est le plus souvent d'origine appendiculaire, mais une origine ovarienne reste probable. En absence du traitement précoce, le pronostic de cette maladie reste sombre. L'amélioration du pronostic passe par un diagnostic précoce des tumeurs mucineuses avant même le stade de l'ascite.

## Conflits d’intérêts

Les auteurs ne déclarent aucun conflit d'intérêts.
